# IL-12p35 Inhibits Neuroinflammation and Ameliorates Autoimmune Encephalomyelitis

**DOI:** 10.3389/fimmu.2017.01258

**Published:** 2017-10-05

**Authors:** Jin Kyeong Choi, Ivy M. Dambuza, Chang He, Cheng-Rong Yu, Anita N. Uche, Mary J. Mattapallil, Rachel R. Caspi, Charles E. Egwuagu

**Affiliations:** ^1^Molecular Immunology Section, Laboratory of Immunology, National Eye Institute (NEI), National Institutes of Health, Bethesda, MD, United States; ^2^State Key Laboratory of Ophthalmology, Zhongshan Ophthalmic Center, Sun Yat-sen University, Guangzhou, China; ^3^Immunoregulation Section, Laboratory of Immunology, National Eye Institute (NEI), National Institutes of Health, Bethesda, MD, United States

**Keywords:** biologic, multiple sclerosis, experimental autoimmune encephalomyelitis, IL-12p35, Breg cells, Treg cells, STATs, cytokine signaling

## Abstract

Multiple sclerosis (MS) is an inflammatory demyelinating disease in which cytokines produced by immune cells that infiltrate the brain and spinal cord play a central role. We show here that the IL-12p35, the alpha subunit of IL-12 or IL-35 cytokine, might be an effective biologic for suppressing neuroinflammatory responses and ameliorating the pathology of experimental autoimmune encephalomyelitis (EAE), the mouse model of human MS. We further show that IL-12p35 conferred protection from neuropathy by inhibiting the expansion of pathogenic Th17 and Th1 cells and inhibiting trafficking of inflammatory cells into the brain and spinal cord. In addition, *in vitro* exposure of encephalitogenic cells to IL-12p35 suppressed their capacity to induce EAE by adoptive transfer. Importantly, the IL-12p35-mediated expansion of Treg and Breg cells and its amelioration of EAE correlated with inhibition of cytokine-induced activation of STAT1/STAT3 pathways. Moreover, IL-12p35 inhibited lymphocyte proliferation by suppressing the expressions of cell-cycle regulatory proteins. Taken together, these results suggest that IL-12p35 can be exploited as a novel biologic for treating central nervous system autoimmune diseases and offers the promise of *ex vivo* production of large amounts of Tregs and Bregs for immunotherapy.

## Introduction

Multiple sclerosis (MS) is a complex inflammatory demyelinating and degenerative disease thought to be triggered by blood-borne leukocytes that invade the central nervous system (CNS). These cells produce inflammatory cytokines such as IL-17 and IFN-γ that act on CNS-resident cells (microglia and astrocytes) to elicit production of additional cytokines (IL-1, IL-6, IL-12, IL-23, and TNF-α) and chemokines that promote further recruitment of leukocytes, which fuel the inflammatory cascade (1, 2). Besides the major histocompatibility complex loci, cytokines are among the most associated risk factor genes for MS and therefore a major target of disease-modifying therapies (immune suppressants, steroids, immune modulators, and biologics) for the treatment of MS (3). Experimental autoimmune encephalomyelitis (EAE) is the prototypical animal model of MS. Much of what we have learned about the pathophysiology of MS has come from EAE, which has also provided valuable insights into therapeutic approaches that might be effective in MS (4). EAE is based on the CNS-extrinsic/peripheral pathogen-induced molecular mimicry model of MS etiology (5–7). Emulsified CNS antigen is administered along with complete Freund’s adjuvant (CFA) (immune stimulants). Autoreactive Th1 and Th17 cells activated at peripheral sites traffic from the draining lymph nodes, cross the blood–brain or blood–cerebrospinal fluid barrier and mediate CNS pathology (1, 4). The requirement of pathogen-associated molecular patterns (PAMPs) contained in CFA for induction of EAE has led to interest in pathways that regulate cytokines elicited in response to PAMPs (8). Regarding potential biologics for MS, the IL-12/IL-6 family of cytokines are of significant interest as they regulate the initiation, intensity, and duration of immune responses and are direct targets of TLR agonists present in CFA (8).

There are currently four known members of this family, IL-12, IL-23, IL-27, and IL-35 (9–12). Each member is composed of two subunits; an alpha subunit structurally similar to the IL-6 superfamily cytokines (p19, p28, and p35) and a beta subunit homologous to type 1 cytokine receptors (p40 and Ebi3) (9). Published reports suggest that the predominant IL-12 family member produced within the environment of differentiating naïve lymphocytes significantly influences their developmental decisions and might therefore determine the lymphocyte subsets that would dominate the ensuing immune response (8). There is now consensus that IL-12 and IL-23 are pro-inflammatory while IL-35 is immune suppressive and a prime candidate as a biologic that can be used to suppress autoimmune diseases (9). That IL-35 is of critical importance in ameliorating CNS autoimmune diseases was demonstrated by several studies indicating that IL-35 can induce the expansion of Treg cells and a unique IL-35-producing regulatory B cell (i35-Breg) population (9, 13–15). However, isolating or producing sufficient quantities of the functional IL-35 heterodimeric cytokine has been challenging and very labor intensive (16, 17). Thus far, this has been a major impediment for *ex vivo* production of large scale IL-35-producing Bregs for adoptive Breg therapy.

An important question concerning the immunobiology of IL-35 relates to the relative contributions of IL-12p35 or Ebi3 subunit to the biological function of IL-35. Specifically, it is unclear whether single chain IL-12p35 or Ebi3 also possesses intrinsic immune-regulatory activities that can be exploited therapeutically. In this study, we have produced and used recombinant IL-12p35 (rIL-12p35) to directly examine whether IL-12p35 possesses some of the immune-suppressive activities attributed to IL-35 and if it can be used as a biologic to suppress EAE, thereby circumventing the arduous task of bioengineering functional recombinant heterodimeric IL-35 for use in Breg therapy.

## Materials and Methods

### Animals

Wild-type C57BL/6J mice were purchased from Jackson Laboratory. All protocols were approved by the NEI Animal Care and Use Committee and followed NIH guidelines for using animals in intramural research.

### Production and Characterization of Mouse rIL-12p35 or p35

Mouse rIL-12p35 construct was generated by RT-PCR using forward primer: 5′-CGCGGATCCATTGGCCAGGGTCATTCCAGT-3′ and reverse primer: 5′-CCGCT CGAGGGCGGAGCTCAGATAG-3′. The IL-12p35 cDNA was cloned into the 3.6 kb pMIB vector containing an amino-terminal honeybee melittin (HBM) secretion signal sequence and a poly-histidine tag to facilitate isolation and characterization, and expression of the recombinant protein was driven by baculovirus immediate-early promoters of the polyhedrosis virus (Catalog # V8030-01; Invitrogen, Carlsbad, CA, USA). The expression construct was transfected into High Five insect cells, and stable transfectants were identified by drug selection (Blasticidin S; 100 µg/ml). To ensure that the recombinant clones expressed *bona fide* rIL-12p35, we isolated the expression vector (HBM-p35-Flag-His) from the stable clones and verified by DNA sequencing that no mutations were introduced during cloning or drug selection. The rIL-12p35 secreted in the insect cell culture was sequentially purified using Ni-NTA Purification system (Invitrogen), size-exclusion centricon filtration and two consecutive cycles of fast performance liquid chromatography (FPLC) gel filtration chromatography. The rIL-12p35 was further characterized by SDS-PAGE, Western blot/immunoprecipitation, and sedimentation equilibrium ultracentrifugation. Authenticity of the protein was confirmed by mass spectroscopy.

### Induction of EAE

Experimental autoimmune encephalomyelitis was induced by subcutaneous immunization with 200 µg myelin oligodendrocyte glycoprotein peptide 35–55 (MOG_35–55_) (Sigma, St. Louis, MO, USA) in CFA emulsion, containing 2.5 mg/ml of heat killed, pulverized *Mycobacterium tuberculosis* strain H37RA. The mice also received two doses of 200 ng *Bordetella pertussis* toxin (Sigma, St. Louis, MO, USA) on day 0, and day 2 post-immunization intraperitoneally (i.p.) injection in 100 µl of RPMI 1640 medium containing 0.1% normal mouse serum. Some mice received p35 (100 ng/mouse) concurrent with immunization with MOG and every other day until day 14 post-immunization. The control or p35-treated group (*n* = 12) was euthanized 21 days post-immunization. The mice were monitored, and disease severity was assessed daily by a masked observer. Clinical signs of EAE were graded according to the following scale: 0, no clinical symptoms; 1, clumsiness, incontinence or atonic bladder, flaccid tail; 2, mild paraparesis (trouble initiating movement); 3, moderate paraparesis (hind limb weakness); 4, complete front and hind limb paralysis; 5, moribund state (18). Spinal cord and brain were harvested 21 days post-immunization and stained with H&E. For adoptive transfer studies, mice with EAE were sacrificed on day 17 post-immunization and used as donors in passive induction of EAE by adoptive transfer of encephalitogenic cells. Spleen and LN cells were isolated, stimulated with MOG_35–55_ peptide (20 µg/ml) for 3 days in the presence or absence of p35 and transferred i.v. to naive syngeneic recipient mice (10 × 10^6^ cells/mouse; *n* = 12). Ten days after adoptive cell transfer, disease was assessed and brain or spinal cord tissue was collected from recipient mice, fixed in 10% buffered formalin and sectioned for histopathological examination (18). CNS infiltrates were collected from the brain and spinal cord, and lymphocytes/mononuclear cells were isolated by percoll gradient for analysis.

### *In Vivo* Model of LPS-Induced Inflammation

C57BL/6J mice were injected with LPS (15 μg/mouse), and some mice received p35 (100 ng/mouse) 1 h before LPS injection by i.p. route. The control or p35-treated group (*n* = 5) was euthanized 96 h post-injection, and spleen cells were subjected to FACS analysis.

### Lymphocyte Proliferation Assay

B cells were stimulated with LPS (1 µg/ml) while CD4^+^ T cells were cultured in plate-bound anti-CD3 antibody (Ab) (3 µg/ml) and medium containing anti-CD28 Ab (1 µg/ml). B cells or T cells were propagated in presence or absence of p35. After 72 h, cultures were pulsed with ^3^H-thymidine (0.5 μCi/10 μl/well) as described previously (13). Presented data are mean CPM ± SEM of responses of five replicate cultures.

### Detection of Cytokine-Expressing Lymphocytes by FACS

Primary CD19^+^ B cells (>98%) isolated from the spleen/LN (sorted for CD19^+^) were stimulated with LPS (1 µg/ml) for 2 days. CD4^+^ T cells (>98%) from the spleen and/or LN were activated in plate-bound anti-CD3 Abs (3 µg/ml) and soluble anti-CD28 Abs (1 µg/ml) as described previously (13). For intracellular cytokine detection, cells were restimulated for 5 h with PMA (20 ng/ml)/ionomycin (1 µM). GolgiStop was added in the last hour, and intracellular cytokine staining was performed using BD Biosciences Cytofix/Cytoperm kit as recommended (BD Pharmingen, San Diego, CA, USA). FACS analysis was performed on a MACSQuant analyzer (Miltenyi Biotec, San Diego, CA, USA) using protein-specific monoclonal antibodies and corresponding isotype control Abs (BD Pharmingen, San Diego, CA, USA) as described previously (13, 19). FACS analysis was performed on samples stained with mAbs conjugated with fluorescent dyes, and each experiment was color-compensated. Dead cells were stained with dead cell exclusion dye (Fixable Viability Dye eFluor^®^ 450; eBioscience), and live cells were subjected to side scatter and forward scatter analysis. Quadrant gates were set using isotype controls with less than 0.2% background.

### Western Blotting Analysis

Preparation of whole cell lysates and performance of Western blot analysis were as described previously (20). Cell extracts (20–40 μg/lane) were fractionated on 4–12% gradient SDS-PAGE, and antibodies used were: pSTAT1, pSTAT3, and pSTAT4 (Cell Signaling Technology); IL-12p35, Ebi3, Cyclin E, cyclin D1, p27^Kip1^, and β-actin (Santa Cruz Biotechnology, Santa Cruz, CA, USA). Pre-immune serum was used in parallel as controls, and signals were detected with HRP-conjugated secondary F(ab′)_2_ Ab (Zymed Laboratories) using the ECL-PLUS system (Amersham, Arlington Heights, IL, USA).

### Statistical Analysis

Statistical analysis was performed by Student’s *t*-test (two-tailed). EAE scores were analyzed by nonparametric Mann–Whiney *U* test (two-tailed). Asterisks denote *p* value (*p < 0.05, **p < 0.01, ***p < 0.001, ****p < 0.0001).

## Results

### IL-12p35 (p35) Reduces the Severity of EAE

The function of IL-12p35 *in vivo* is unknown, and loss of IL-12p35 in mouse models of autoimmune disease has produced conflicting results. This is in part because IL-12p35 is a subunit of IL-12 and IL-35, two IL-12 family cytokines that exert opposite effects on inflammatory responses. While IL-12p35-deficient mice are protected against collagen-induced arthritis (21), these mice develop exacerbated EAE (22). In this study, we produced the mouse rIL-12p35 and used it to further investigate its potential *in vivo* functions and to examine whether it might possess intrinsic immune-suppressive effects that can be explored therapeutically. The rIL-12p35 was produced in insect cells, and the secreted protein was sequentially purified using Ni-NTA Purification system (Invitrogen), size-exclusion Centricon filtration, and two consecutive cycles of FPLC gel filtration chromatography (Figure S1 in Supplementary Material). The purified rIL-12p35 (p35) was characterized by SDS-PAGE, Western blot/immunoprecipitation, and sedimentation equilibrium ultracentrifugation and was found to exhibit a molecular weight of ~27 kDa on reduced SDS gel (Figure S1 in Supplementary Material).

**Figure 1:**
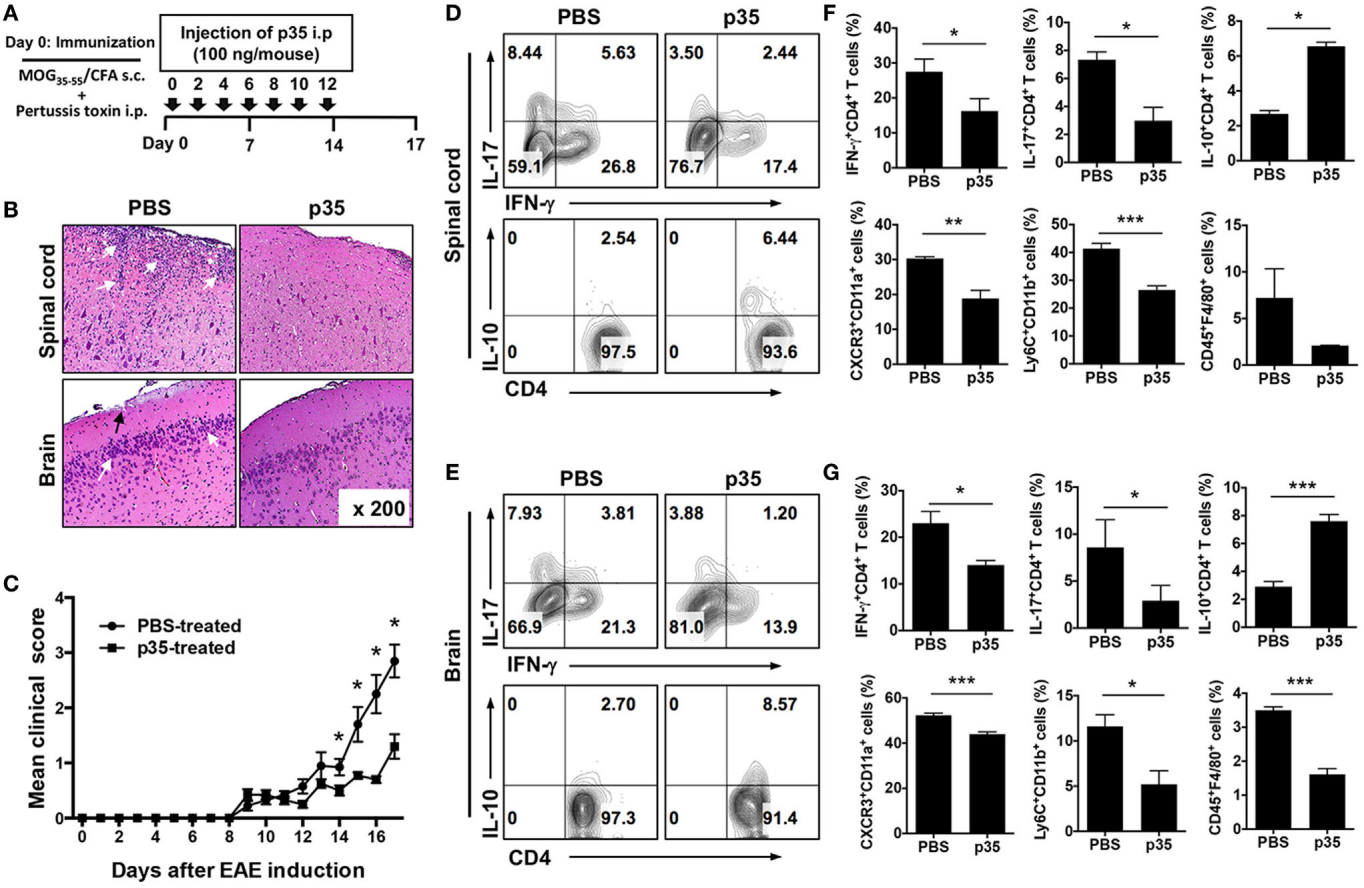
IL-12p35 reduced the severity of experimental autoimmune encephalomyelitis (EAE) induced by active immunization with MOG_35–55_. **(A)** Schematic showing the treatment strategy. EAE was induced by immunization of C57BL/6J mice treated with MOG_35–55_-peptide in complete Freund’s adjuvant (CFA) (*n* = 12). Mice were treated by intraperitoneal injection of IL-12p35 (100 ng/mouse) or PBS on day 0 of immunization and every other day until day 12 post-immunization. **(B)** Representative photomicrographs of H&E stained sections of spinal cord and brain of mice on day 17 post-immunization (original magnification 200×). White arrows show inflammatory cells in the spinal cord or brain; black arrows denote inflammatory lesions in brain subpial location. **(C)** EAE clinical scores. **(D–G)** Characterization of immune responses on IL-12p35-treated EAE mice. Both spinal cord and brain cells were collected from individual mice, and the isolated cells were analyzed using FACS. The data are presented as the mean ± SEM of three determinations. ****p < 0.0001; ***p < 0.001; **p < 0.01; *p < 0.05, significantly different from PBS-treated EAE.

To directly examine whether p35 might possess immune-suppressive activities *in vivo*, we induced EAE in C57BL/6J mice by active immunization with MOG_35–55_ peptide in CFA and administered p35 concurrently with immunization as indicated in Figure 1A. Disease progression and severity were monitored as described in the Section “Materials and Methods.” Consistent with published reports, untreated mice developed severe EAE characterized by infiltration of inflammatory cells into the brain and spinal cord (Figure 1B), flaccid tail, paraparesis, complete front and hind limb paralysis, and/or moribund state. These hallmark features of encephalitis were much reduced in mice treated with p35, as indicated by the reduced disease score (Figure 1C). Analysis of histopathologic sections of the immunized mice revealed substantial infiltration of inflammatory cells into the spinal cord and brain of the untreated mice with EAE compared with a marked reduction of infiltrated inflammatory cells into these tissues of the p35 treatment group (Figure 1B).

The recruitment of IL-17-producing Th17 cells, IFN-γ-expressing Th1 lymphocytes as well as several other inflammatory cells of the myeloid lineage into the brain and spinal cord are implicated in the immunopathogenic mechanisms that contribute to MS and EAE (22, 23). We therefore isolated cells from the brain and spinal cord and examined whether p35-reduced EAE severity correlated with a reduction of these pathogenic lymphoid and myeloid cell types in the CNS. In line with our prediction, frequency of pathogenic Th17 and Th1 cells was significantly reduced in both the spinal cord and brain of p35-treated mice compared with untreated mice and interestingly, this was accompanied by increase of IL-10-expressing CD4^+^ T cells (Figures 1D,E). In EAE, infiltrating CD4^+^ T cells are reactivated in the CNS by antigen-presenting cells, leading to exacerbation of the neuroinflammatory responses, enhanced production of inflammatory cytokines by microglia/astrocytes and further recruitment of monocytes into the CNS (2). Analysis of the brain and spinal cord revealed recruitment of inflammatory “monocyte-derived” myeloid cells characterized by CD11b^+^Ly6C^+^ phenotype, which were significantly reduced in the brain or spinal cord of the p35-treated mice (Figures 1F,G). Of note, these inflammatory monocyte-derived myeloid cells are rare in normal mice but increase during inflammation, and their disappearance correlates with disease resolution (24). Furthermore, diminished recruitment of the inflammatory cells into the brain or spinal cord of the p35-treated mice correlates with reduced expression of CXCR3 (Figures 1F,G), suggesting that p35 might mitigate EAE, in part, by suppressing trafficking of inflammatory cells into the CNS.

### IL-12p35 Induces Expansion of IL-35-Expressing Treg and Breg Cells

**Figure 2:**
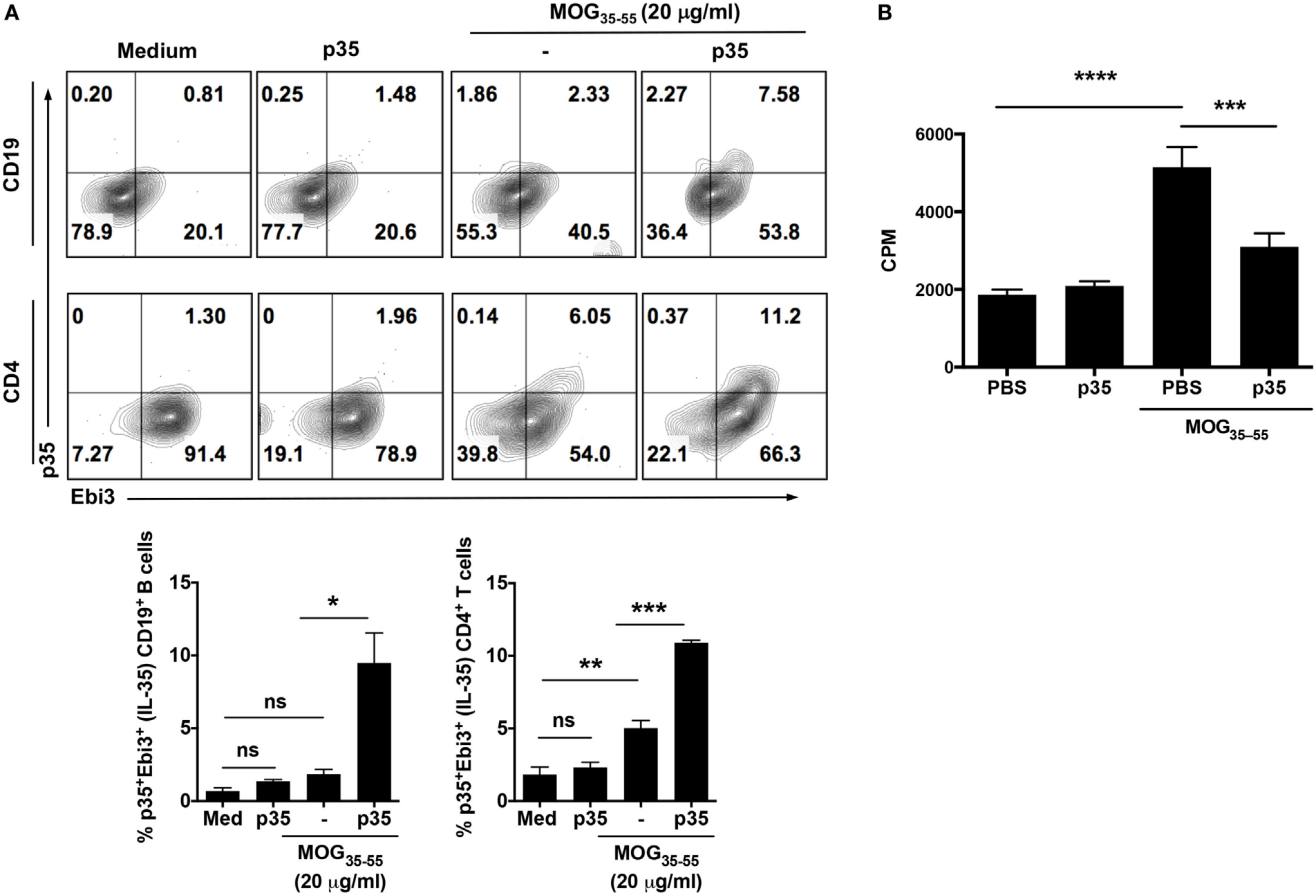
IL-12p35 induces expansion of IL-35-expressing B cells in the spleen during experimental autoimmune encephalomyelitis (EAE). **(A)** EAE was induced in mice by active immunization with MOG_35–55_-peptide in complete Freund’s adjuvant, and the mice were treated with IL-12p35 or PBS. Spleen cells isolated from the mice on day 17 after EAE induction were restimulated *ex vivo* with MOG_35–55_ in presence or absence of IL-12p35 for 72 h and were analyzed by intracellular cytokine staining analysis **(A)** or lymphocyte proliferation assay **(B)**. **(A)** For intracellular cytokine assay, cells were gated on CD19^+^ or CD4^+^ cells, and numbers in the quadrants indicate the percentages of T or B cells expressing IL-35 (p35 and Ebi3). The data are presented as the mean ± SEM of three determinations. ****p < 0.0001; ***p < 0.001; **p < 0.01; *p < 0.05, significantly different from PBS-treated. **(B)** Proliferative responses assessed by ^3^H-thymidine incorporation assay were analyzed in five replicate cultures, and data presented as mean value of CPM of the five replicate cultures. Results represent at least three independent experiments. *p < 0.05, **p < 0.01, ***p < 0.001, ****p < 0.0001 [Student’s *t*-test (two-tailed)].

The heterodimeric IL-35 cytokine, of which p35 is a subunit, has previously been shown to inhibit expansion of pathogenic Th17 cells and to play a critical role in mitigating autoimmune diseases by inducing expansion of IL-10 and/or IL-35-expressing Breg cells (13, 15). We therefore investigated whether suppression of pathogenic Th17 responses and amelioration of EAE mediated by p35 derived in part from inducing expansion of regulatory cells in the B cell compartment. Mice were immunized with the MOG_35–55_ peptide and treated with p35 or PBS. Control mice received CFA alone. Seventeen days post-immunization, cells harvested from the spleen of PBS-treated or p35-treated mice were restimulated *in vitro* with MOG_35–55_ for 72 h. Intracellular cytokine staining revealed that p35-induced significant expansion of IL-35-producing B cells in the spleen compared with PBS-treated mice (Figure 2A; top panel). Surprisingly, we also observed significant expansion of IL-35-producing T cells in the spleen of the p35-treated mice (Figure 2A; lower panel). This observation is consistent with previous studies showing that IL-35 can induce the expansion of a regulatory T cell population that mediates immune suppression *via* IL-35 (25). Results of the lymphocyte proliferation assay further show that the increase of these regulatory lymphocyte subsets in the spleen of p35-treated mice correlates with suppression of MOG-specific encephalitogenic T cells (Figure 2B).

### IL-12p35 Promotes Expansion of Treg Cells in the Brain and Spinal Cord during EAE

**Figure 3:**
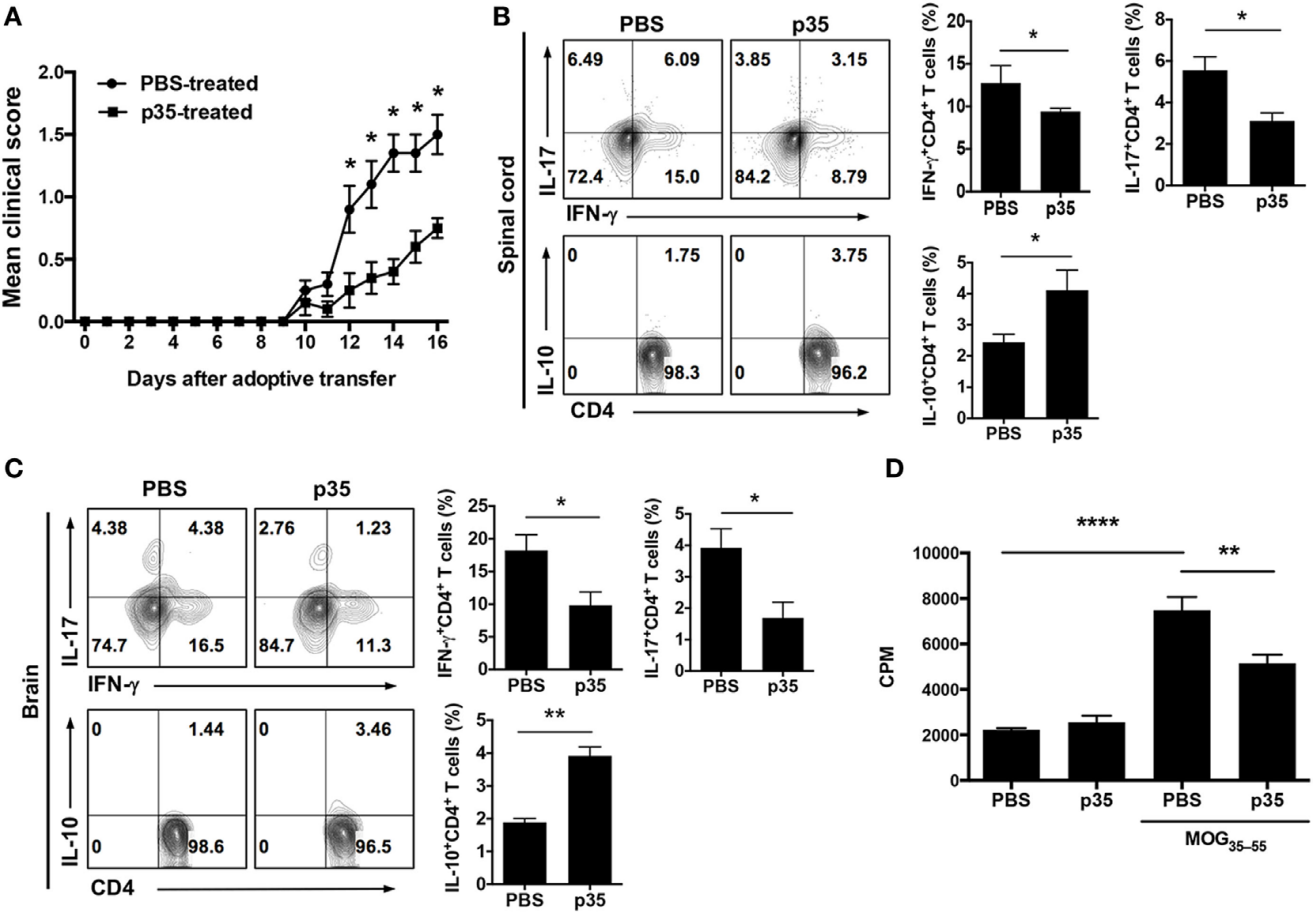
IL-12p35 treatment suppressed adoptive transfer of experimental autoimmune encephalomyelitis (EAE) by encephalitogenic T cells. EAE was induced in mice by active immunization with MOG_35–55_-peptide in complete Freund’s adjuvant, and the mice were treated with p35 or PBS. Spleen cells from the mice on day 17 after EAE induction were restimulated *ex vivo* with MOG_35–55_ for 72 h. Activated cells (1 × 10^7^ cells/mouse) were transferred to naïve syngeneic mice and clinical disease monitored until day 17 days after adoptive transfer of encephalitogenic cells. **(A)** EAE clinical scores. **(B,C)** On day 17 after adoptive transfer, cells were isolated from the brain or spinal cord and analyzed by intracellular cytokine staining. Cells were gated on CD4^+^ cells, and numbers in quadrants indicate percentage of cells IFN-γ-, IL-17-, and IL-10-expressing CD4^+^ T cells. **(D)** Proliferative responses of cells in the spinal cord and brain of C57BL/6J recipient mice 17 days after adoptive transfer were assessed by ^3^H-thymidine incorporation assay. Five replicate cultures were analyzed, and data presented as mean value of CPM of the five replicate cultures. The data are presented as the mean ± SEM of three determinations. ****p < 0.0001; ***p < 0.001; **p < 0.01; *p < 0.05, significantly different from adoptive transfer of MOG_35–55_-stimulated with PBS.

The observation that p35 can inhibit EAE induced by active immunization with MOG peptide/CFA was interesting, however, for targeted therapy, it was important to determine whether the capacity of encephalitogenic T cells to induce EAE is compromised in mice treated with p35. For this purpose, we isolated cells from the spleen and LN of mice with EAE or from EAE mice that were treated with p35. After *in vitro* expansion with MOG peptide, 10 × 10^6^ cells were transferred into naïve syngeneic mice, and the animals were monitored over 16 days for the development of EAE (as described in the Section “Material and Methods”). As indicated by the EAE clinical scores, cells derived from PBS-treated EAE mice efficiently transferred disease while mice that received cells from p35-treated EAE mice induced significantly milder disease (Figure 3A). Intracellular cytokine analysis of cells isolated from spinal cord and brain suggested that p35 suppressed the capacity of pathogenic Th1 and Th17 cells to induce EAE by promoting the expansion of IL-10-producing T cells (Figures 3B,C). We also analyzed the effect of p35 treatment on encephalitogenic cells derived from the spleen and brain by lymphocyte proliferation assay. The data revealed that the increase of regulatory lymphocyte subsets in the spinal cord, brain, and spleen of the p35-treated mice was accompanied by significant suppression of MOG-specific encephalitogenic cells during EAE (Figure 3D). Taken together, these results suggest that *in vivo* exposure to p35 can be used to attenuate the pathogenic potential of encephalitogenic T cells, and that this might derive at least in part from the capacity of p35 to induce the expansion of IL-10-producing CD4^+^ T cells.

### IL-12p35 Inhibits Cytokine-Induced STAT Pathways and Cell-Cycle Regulatory Proteins

**Figure 4:**
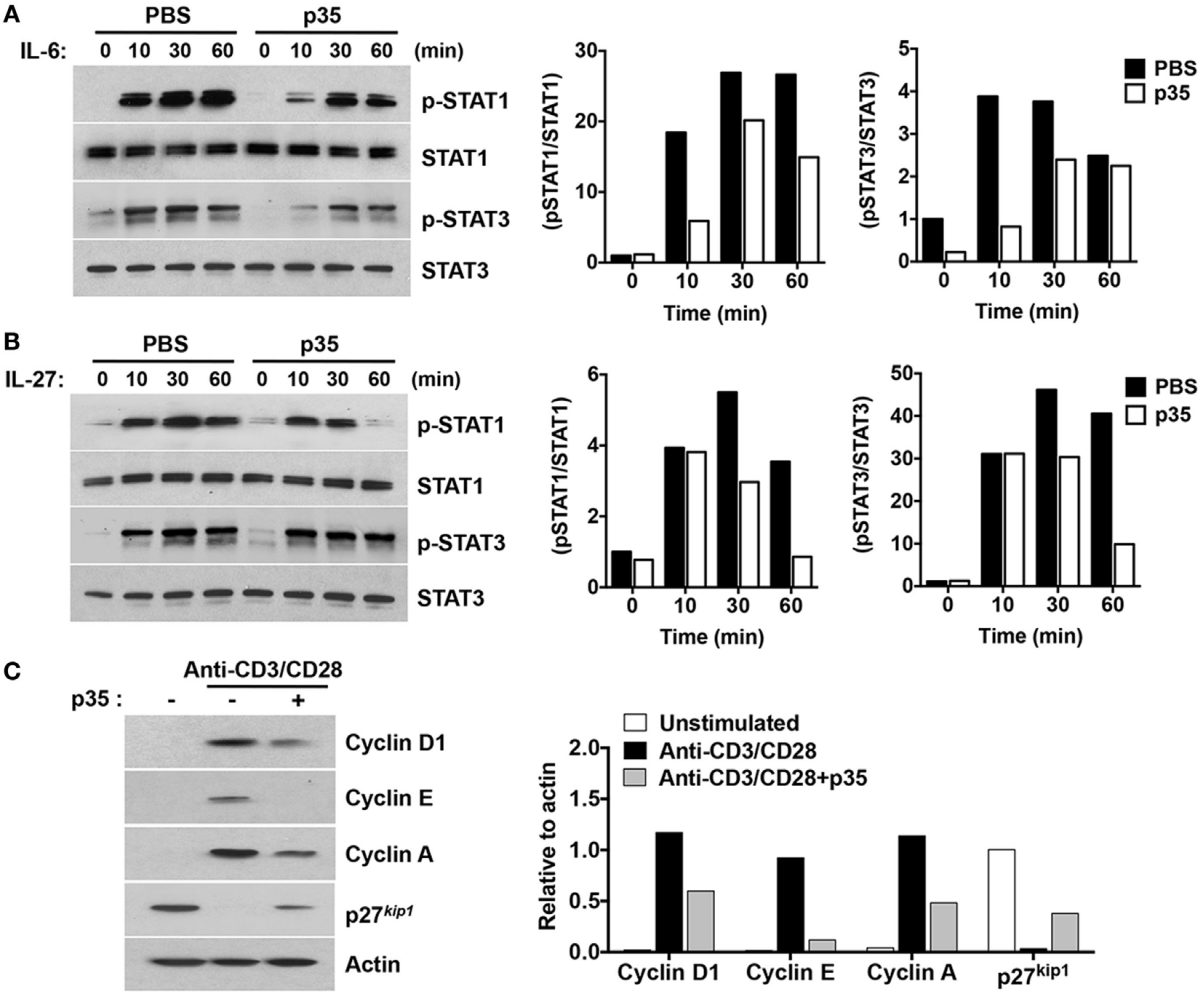
IL-12p35 inhibits STAT signaling pathway and cell-cycle regulatory proteins. **(A,B)** CD4^+^ T cells were stimulated in anti-CD3 coated plates and with anti-CD28 for 72 h. The cells were then washed, cultured in serum-free medium with p35 for 2 h, followed by stimulation with IL-6 or IL-27 for the indicated time periods. **(C)** Purified CD4^+^ T cells were TCR activated for 48 h as described earlier. Whole cell lysates were analyzed by Western blotting. Protein levels were normalized to total STAT or β-actin and quantified using Image-J software. Data are representative of at least three experiments.

Antigen-presenting dendritic cells (DCs) influence the differentiation, proliferation, and effector functions of adaptive immune cells *via* secretion of cytokines such as IL-6 and/or IL-27. IL-6 regulates T cells through activation of STAT3 (9, 26–30) whereas IL-27 regulates inflammation through activation of STAT1 and STAT3 pathways (12). We therefore investigated whether, mechanistically, p35 might have mediated EAE by inhibiting cytokine-induced activation of STAT1 and STAT3 downstream of gp130, a common receptor utilized by both cytokines. CD4^+^ T cells were isolated from C57BL/6J mice and stimulated for 72 h in anti-CD3 coated plates (3 µg/ml) containing anti-CD28 (1 µg/ml). After 72 h, the cells were starved for 2 h in serum-free medium containing 0.5% BSA and stimulated for 0–60 min in culture medium containing IL-6 or IL-27 (10 ng/ml). Western blot analysis revealed that unlike the heterodimeric IL-35, p35 could not activate the STAT1 or STAT3 pathway. Rather, it inhibited activation of both STAT1 and STAT3 pathways (Figures 4A,B). These observations are in line with a recent study showing that while IL-27p28 by itself could not activate the STAT pathway, it antagonized gp130-mediated signaling (31). Western blot analysis of the cells cultured for 3 days also revealed that p35 might also mediate its suppressive activities by antagonizing cell-cycle proteins that regulate lymphocyte proliferation (Figure 4C). Taken together, the p35-mediated suppression of pathogenic Th17 cells and its amelioration of EAE correlate with inhibition of cytokine-induced activation of STAT1/STAT3 pathways and perturbation of cell-cycle events that regulate lymphocyte expansion.

### IL-12p35 and IL-35 Activate Overlapping and Distinct Immune-Regulatory Mechanisms

**Figure 5:**
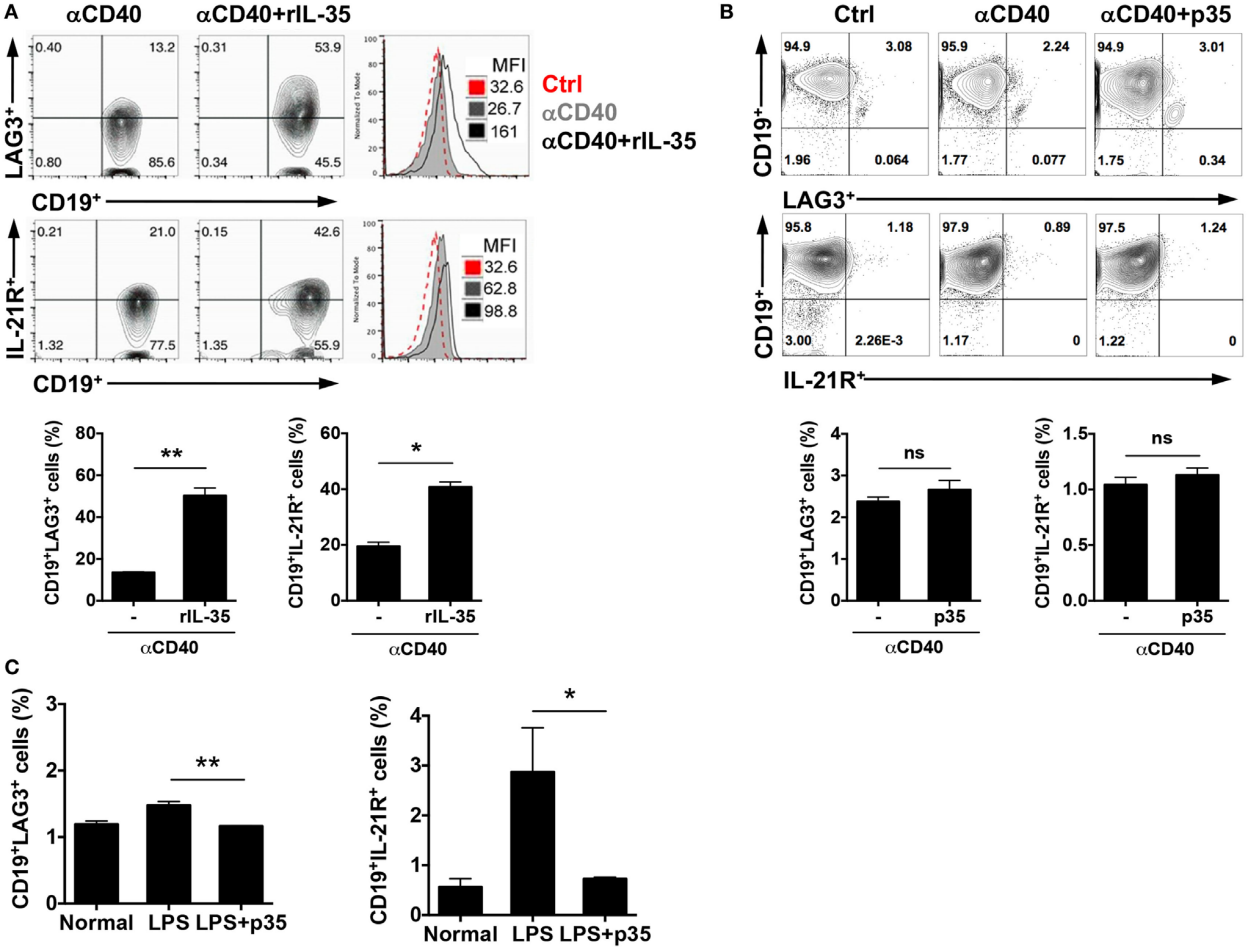
IL-35 but not IL-12p35 induces expansion of LAG3- and IL-21 receptor (IL-21R)-expressing B cells. **(A)** Sorted CD19^+^ B cells were cultured in the absence or presence of anti-CD40Ab (2 µg/ml) or anti-CD40 antibody (Ab) plus IL-35 (100 ng/ml) for 4 days. **(B)** Sorted CD19^+^ B cells were cultured in the absence or presence of anti-CD40Ab or anti-CD40 Ab plus p35 (100 ng/ml) for 4 days. The expression of LAG3 and IL-21R were analyzed with FACS. **(C)** Mice were injected with LPS (15 μg/mouse) *via* i.p. After 96 h, we analyzed the expression of LAG3 or IL-21R by FACS. The data are presented as the mean ± SEM of four determinations. ****p < 0.0001; ***p < 0.001; **p < 0.01; *p < 0.05. Data are representative of at least three independent experiments.

Although the mechanism(s) by which IL-35 mediates its biological activities are not fully understood, data provided here suggest that IL-12p35 single chain subunit recapitulate some of the published immune-suppressive effects attributed to IL-35. However, there are mechanistic differences between IL-12p35 and IL-35 heterodimeric cytokine that derive in part from the fact that IL-35 mediates its biological effects through activation of STAT1 and STAT3 while IL-12p35 could not activate these STAT pathways (Figure 4). Rather, IL-12p35 seems to function by antagonizing STAT pathways. Surprisingly, we have uncovered in this study two immune-regulatory molecules, LAG3 and IL-21 receptor (IL-21R), that are differentially expressed by activated B cells in response to IL-35 or IL-12p35 stimulation (Figure 5). In response to *in vitro* stimulation of sorted CD19^+^ B cells in medium containing anti-CD40 and/or IL-35, we observed significant increase in the frequency of B cells expressing LAG3 or IL-21R compared with cells cultured in medium containing anti-CD40 (Figure 5A). In contrast to the effects of IL-35 on the expression of LAG3 or IL-21R, culturing CD19^+^ B cells in medium containing anti-CD40 and p35 had no effect on the percentage of B cells expressing either protein (Figure 5B). We next examined whether these observations also pertain to B cells activated *via* T-independent pathway, such as by LPS. Similarly, activation of CD19^+^ cells with LPDS and/or p35 did not induce expansion of LAG3- and/or IL-21R-expressing B cells, underscoring an additional difference that might contribute to differential effects of p35 and IL-35 on B cells (Figure 5C).

## Discussion

There is increasing awareness that microorganisms and microbial products might be important triggers for autoimmune and chronic inflammatory diseases. Thus, PAMPs that act through TLRs on DC lead to the production of pro-inflammatory IL-12 family cytokines as well as suppressive members of the family that guard against excessive immune responses that cause autoimmune pathology. While these heterodimeric cytokines are under intense investigation, less attention has been given to the physiological functions of the individual single chain proteins that are themselves differentially regulated by pathways downstream of distinct adaptor molecules that mediate transcriptional programs activated by TLRs.

In this study, we have shown that the IL-12p35 subunit protein possesses intrinsic immune-suppressive activities during EAE and can therefore be exploited as a biologic for the treatment of CNS autoimmune diseases. IL-12p35 suppressed lymphocyte proliferation, antagonized pathogenic Th17 responses, and ameliorated encephalitis in the EAE model by promoting the expansion of Tregs as well as IL-10- and IL-35-producing Breg cells in the brain and spinal cord. We further show that exposure of pathogenic encephalitogenic T cells to IL-12p35 rendered the cells partially anergic, resulting in diminished capacity to transfer EAE to naïve syngeneic mice. Importantly, IL-12p35 induced the expansion of IL-10-producing CD4^+^ T cells in the brain and spinal cord of mice treated with p35. Furthermore, the increases in regulatory cells in the brain or spinal cord correlated with decrease in the recruitment of Th17/Th1 cells and inflammatory myeloid cells into these tissues and lower EAE clinical scores. Thus, p35 treatment recapitulates some of the suppressive effects exhibited by IL-35 in CNS autoimmune diseases.

In context of the mechanism by which IL-12p35 mediates its immune-suppressive activities, we observed some mechanistic difference between IL-12p35 and the heterodimeric IL-35 cytokine. While IL-35 mediates its biological effects through activation of STAT1, STAT4, and STAT3, IL-12p35 did not activate STAT proteins but instead antagonized STAT pathways induced by IL-6 and IL-27. These observations are reminiscent of observation reported for IL-27p28, one of the subunits of IL-27 (29). IL-27p28, independently of Ebi3, antagonized cytokine signaling through gp130 (29). Notably, IL-27p28 treatment was able to inhibit pathogenic Th1 and Th17 responses and provide protection from EAE as well as from experimental autoimmune uveitis, an autoimmune disease model that shares essential immunological mechanisms with EAE (31). Furthermore, mice transgenic for IL-27p28 expression exhibit defects in germinal center formation and Ab production, further supporting the notion that single chain IL-12 family cytokines might possess intrinsic anti-inflammatory activities that can be exploited therapeutically. Interestingly, the heterodimeric IL-27 (p28/Ebi3) is thought exerts mostly anti-inflammatory roles in EAE through the induction of IL-10 in the CNS suggesting that the p35-mediated inhibition of IL-27 signaling may not completely explain the ability of p35 to ameliorate EAE and increase IL10. It is, however, of note that IL-27 can exert diametrically opposite effects on host immunity deriving from its differential effects on naïve and mature T cells: it promotes the differentiation of naive CD4^+^ T cells into pathogenic Th1 cells while suppressing production of pro-inflammatory cytokines by mature CD4^+^ T-helper cells through the induction of IL-10. Discerning the physiological effects of IL-27 is further complicated by the fact that IL-27 activates both STAT1 and STAT3 in early activated CD4^+^ T cells while preferentially activating STAT3 in mature CD4^+^ T cells. A consequence of p35-mediated suppression of IL-27-induced activation of STAT1 might be to antagonize the differentiation of naïve T cells into pathogenic Th1 cells during antigen priming while p35-mediated suppression of STAT3 activation might deprive the mature Th17 cell of a transcription factor required for its effector functions. Thus, p35-induced suppression of Th1 and Th17 cells might in turn increase the frequency of Treg cells.

Another mechanistic difference between IL-12p35 and IL-35 that might be of functional relevance is their differential capacity to induce the expansion of B cells with upregulated expression of LAG3 and IL-21R. While IL-35 induced the expansion of B cells expressing LAG3 or IL-21R, IL-12p35 could not induce the expansion of B cells that express these important immune-regulatory proteins that mediate immune tolerance and lymphocyte development. Although increased expression of LAG3 on T cells is associated with immune suppression, additional studies are required to understand the functional implication of the upregulation of LAG3 on B cells (32). Nonetheless, the data showing that IL-35 induced the expansion of B cells with increased expression of IL-21R is intriguing in view of a recent report that functional maturation of IL-10-secreting Breg cells requires IL-21-dependent cognate interactions between B and T cells (33).

Despite the significant excitement over the possibility of using IL-35 as a biologic for the treatment of autoimmune disease, its therapeutic use should be approached with caution because of the promiscuous chain pairing exhibited by IL-12 family cytokines. In addition, there is a dearth of pharmacokinetic data on the bioavailability of the IL-35 and the difficulty of ascertaining the stability IL-12p35:Ebi3 complex *in vivo*. Unlike IL-12 or IL-23 that are secreted as covalently bound heterodimer of IL-12p35:IL-12p40 or IL-23p19:IL-12p40, the IL-12p35 and Ebi3 subunits are independently secreted and thought to eventually associate under physiological condition and form the stable IL-12p35:Ebi3 complex that exhibits the immune-suppressive functions attributed to the heterodimeric IL-35 cytokine. Moreover, the factors that promote or regulate the formation and stability of the heterodimer are currently unknown. While prolonged maintenance of the stable IL-12p35:Ebi3 complex is desirable for treating an autoimmune disease, it also poses the risk of suppressing antitumor immune responses or compromising the efficacy of vaccines against infectious diseases. The difficulty of predicting the immunological outcome of off-target pairing of either IL-12p35 or Ebi3 with other alpha/beta single IL-12 subunit proteins such as p19, p28, or p40 is also worrisome, as underscored by the recent discovery of IL-39, a novel pairing of Ebi3 and IL-23p19 that mediates pro-inflammatory responses in Lupus-like mice (34). Despite the fact that similar concerns can also be raised for the use of IL-12p35, it should be borne in mind that other FDA approved biologics for treatment of MS, such as interferon beta-1b, interferon beta-1a, natalizumab, alemtuzumab, fingolimod, or daclizumab are associated with adverse effects but are clinically beneficial if used judiciously. Nonetheless, ease in producing biologically active IL-12p35 and capacity of the p35 to induce regulatory T and B cells is unique, making IL-12p35 a particularly attractive biologics.

In summary, we have found that IL-35-producing T and B cells are critical regulators of autoimmune and infectious diseases, and that IL-35 can convert human/mouse T and B cells into Tregs and Bregs, respectively. However, production of large amounts of biologically active IL-35 for therapeutic use is difficult and labor intensive, thus providing the major impetus to examine whether the IL-12p35 subunit can recapitulate some of the immunosuppressive activities of IL-35. Discovery that IL-12p35 can also induce IL-10 or IL-35-producing Treg/Breg cells offers the promise of Treg and Breg immunotherapy and would facilitate investigations on the role of i35-Bregs and iT_R_35 cells in autoimmune diseases and cancer.

## Ethics Statement

Wild-type C57BL/6J mice were purchased from Jackson Laboratory. Mice were maintained and used in accordance with NEI/NIH Animal Care and Use Committee guidelines (ASP Protocol # EY000262-19 and EY000372-14).

## Author Contributions

JC performed most of the studies. ID purified and characterized rIL-12p35 and rEbi3, conducted EAE experiments, prepared figures, and edited manuscript. CH conducted EAE experiments, prepared the figures, and edited the manuscript. C-RY assisted with FACS analysis. MM assisted with EAE experiments and EAE scoring. AU assisted with isolation of lymphoid cells; RC provided expertise in analysis of EAE experiments and editing manuscript. CE conceived, designed, and supervised the project and wrote the manuscript.

## Conflict of Interest Statement

The authors declare that the research was conducted in the absence of any commercial or financial relationships that could be construed as a potential conflict of interest.

## Supplementary Material

Figure S1 Schematic representation of the cDNA construct used to produce the recombinant IL-12p35 (p35) protein in insect cells. **(A)** HBM, honeybee melittin secretion signal; FLAG and HIS are tags used to facilitate the isolation and purification of the p35 protein. **(B,C)** Characterization of the p35 protein following two sequential purification cycles on fast performance liquid chromatography columns. **(D)** Western blot analysis of the purified p35 protein.

## Abbreviations

MS, multiple sclerosis; EAE, experimental autoimmune encephalomyelitis; i35-Bregs, IL-35-producing regulatory B cells; CNS, central nervous system; MOG_35–55_, myelin oligodendrocyte glycoprotein peptide 35–55; CFA, complete Freund’s adjuvant.
