# Supplementary figures and images for: IL-12p35 Inhibits Neuroinflammation and Ameliorates Autoimmune Encephalomyelitis
Source: Front Immunol. 2017 Oct 5;8:1258. doi: 10.3389/fimmu.2017.01258 (PMC5633738; doi:10.3389/fimmu.2017.01258)

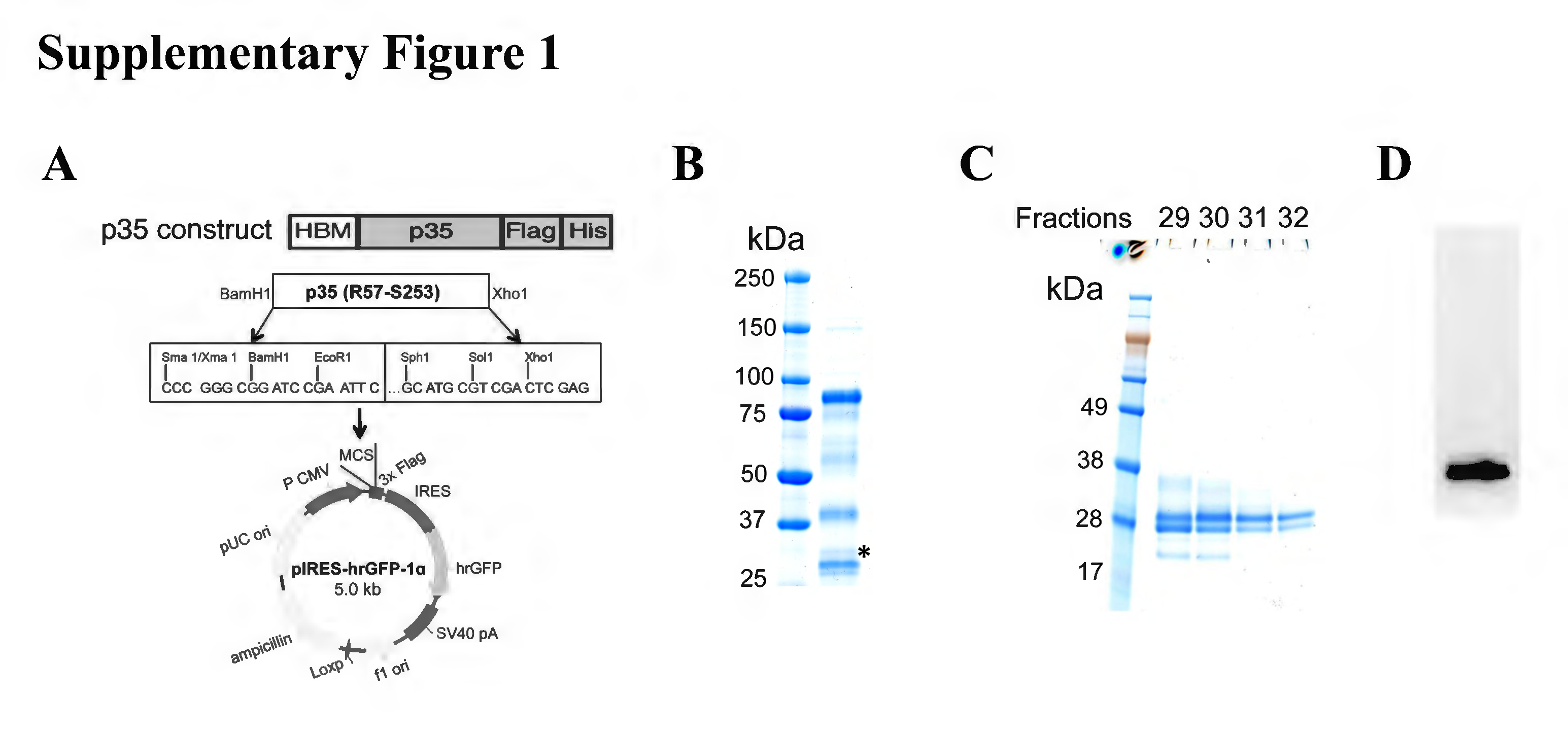

Supplement: Figure S1 — Schematic representation of the cDNA construct used to produce the recombinant IL-12p35 (p35) protein in insect cells. (A) HBM, honeybee melittin secretion signal; FLAG and HIS are tags used to facilitate the isolation and purification of the p35 protein. (B,C) Characterization of the p35 protein following two sequential purification cycles on fast performance liquid chromatography columns. (D) Western blot analysis of the purified p35 protein. [file image_1.tif]
